# Twelve-month effectiveness of telephone and SMS support to mothers with children aged 2 years in reducing children’s BMI: a randomized controlled trial

**DOI:** 10.1038/s41366-023-01311-7

**Published:** 2023-04-22

**Authors:** Li Ming Wen, Huilan Xu, Philayrath Phongsavan, Chris Rissel, Alison Hayes, Sarah Taki, Limin Buchanan, Lisa Simone, Renee Moreton, Louise A. Baur

**Affiliations:** 1 Health Promotion Unit, Population Health Research & Evaluation Hub, Sydney Local Health District, Sydney, NSW Australia; 2 Sydney School of Public Health, Faculty of Medicine and Health, and Charles Perkins Centre, The University of Sydney, Sydney, NSW Australia; 3 NHMRC Centre of Research Excellence in the Early Prevention of Obesity in Childhood (EPOCH), Sydney, NSW Australia; 4 Sydney Institute for Women, Children and Their Families, Sydney Local Health District, Sydney, NSW Australia; 5 College of Medicine and Public Health, Rural and Remote Health, South Australia and Northern Territory, Flinders University, Bedford Park, SA Australia; 6 Population Health, Sydney Local Health District, Sydney, NSW Australia; 7 Specialty of Child and Adolescent Health, Sydney Medical School, The University of Sydney, Sydney, NSW Australia

**Keywords:** Epidemiology, Risk factors

## Abstract

**Background/objectives:**

Few quality intervention studies have assessed whether a combined telephone and short message service (SMS) intervention to mothers is effective in reducing BMI and obesity risk behaviors of children at 3 years of age. This study aimed to assess effectiveness of telephone and SMS support in reducing children’s body mass index (BMI) and obesity risk behaviors.

**Subjects/Methods:**

A randomized controlled trial (RCT) with 662 women of 2-year-old children (with the proportion of overweight and obesity being similar to the general population) was conducted in Sydney, Australia, March 2019–October 2020. The mothers in the intervention group received three telephone support sessions plus SMS messages and mailed-intervention-booklets over a 12 months period i.e., 24–26, 28–30, and 32–34 months of the child’s age. Mothers in the control group received usual care and two mailed booklets on information not related to the intervention. The primary outcome was child’s BMI at 3 years of age. Secondary outcomes were children’s dietary and activity behaviors. All outcome measures were based on mothers’ self-report using standardized tools due to COVID-19 pandemic restrictions.

**Results:**

537 (81%) mothers completed the post-intervention assessment at 3 years with only 470 (71%) children having weight and height measures. Multiple imputation analysis showed no statistically significant difference in mean BMI between the groups. Children in the intervention group were more likely not to eat in front of the TV [AOR 1.79 (95% CI 1.17–2.73), *P* = 0.008], more likely to meet the dietary recommendations [AOR 1.73 (95% CI 0.99–3.02), *P* = 0.054] and meet the activity recommendations [AOR 1.72 (95% CI 1.11–2.67), *P* = 0.015] than those in the control group respectively. Among those with an annual household income (<AUD$80,000), the intervention was significantly associated with a lower mean BMI [16.26 (SD 2.22) kg.m^−2^] in the intervention group than [16.84 (SD 2.37)] in the control, a difference of −0.59 kg/m^2^ (95% CI: −1.15 to −0.03, *P* = 0.040).

**Conclusions:**

A staged telephone and SMS support intervention to mothers with children aged 2 years was associated with improved dietary and activity behaviors. The intervention was also associated with reduced children’s BMI at age 3 years only for those from lower income households.

**Trial registration:**

The trial is registered with the Australian Clinical Trial Registry (ACTRN12618001571268)

## Introduction

Globally, childhood overweight and obesity present a major public health challenge, with prevalence rates having increased substantially over the past four decades [1]. According to World Health Organization estimates, 39 million children under the age of 5 were affected by overweight or obesity in 2020, and over 340 million children and adolescents aged 5–19 had overweight or obesity in 2016 [2]. In Australia, obesity is also a major health burden: one in four (24%) Australian children aged 5–14 years were affected by overweight (17%) or obesity (7.7%) in 2017–18 [3]. The prevalence of overweight and obesity remains higher in those of lower socioeconomic status (SES) [4, 5]. There is evidence linking obesity in childhood to adolescence and adulthood obesity [6], and at least 18 co-morbidities [7]. Thus, preventing obesity and related risk behaviors in early childhood is critical for long-term health outcomes.

To date, evidence for effective early obesity interventions is still developing. A 2019 systematic review showed weak to moderate evidence from 16 randomized controlled trials (RCTs) on reducing the risk of obesity [i.e., body mass index (BMI)] in young children aged 0–5 years [8]. The review also found all early interventions combining diet and physical activity components were delivered through a face-to-face approach in childcare center, community or home settings [8]. However, over the past three years, as a result of the COVID-19 pandemic, governments globally implemented “social distancing” and “self-isolation” and have decreased or suspended many face-to-face health programs and services in an effort to contain the spread of the virus [9]. Therefore, finding effective obesity prevention strategies that provide alternatives to face-to-face services has become pertinent during the pandemic and beyond.

The use of telephone calls or short message service (SMS), which have become increasingly popular due to easy access and low cost, provide innovative opportunities for health promotion programs such as early obesity prevention [10–12]. A recent Australian 3-arm RCT study [13] demonstrated that nurse-led, staged telephone support can be an alternative approach to widely used face-to-face approaches in promoting healthy eating habits and reducing screen time in the first two years of life and appeared to be more effective than SMS support [11, 12]. However, neither telephone nor SMS support alone showed a significant effect on the BMI of participating children at two years of age [12]. The study called for further investigation of the effectiveness of combined telephone and SMS support in reducing BMI in young children since there has been no quality research available in this area.

To fill this knowledge gap, we conducted this trial to test whether a combined telephone and SMS support intervention to parents is effective in reducing child BMI and promoting healthy eating and physical activity at 3 years of age [14].

## Methods

### Study design

We conducted a 2-arm parallel RCT during March 2019 and October 2020 with a three staged nurse-led telephone and SMS support intervention that targeted mothers of children aged 2 years. The study protocol was published prior to the commencement of the study, together with the trial registration [14]. The protocol was implemented with some amendments mainly for stage 3 intervention content (e.g., covering some COVID-19 related information) and measurement of height and weight due to the COVID-19 pandemic prohibiting face-to-face data collection.

### Setting

The study was built directly on the existing 3-arm CHAT trial [13] conducted in metropolitan Sydney, New South Wales (NSW), Australia, with recruitment from antenatal clinics in eight hospitals of four local health districts [11, 12, 15]. Briefly, the existing trial recruited women (*n* = 1155) from late pregnancy with follow up until their children were aged 2 years [11, 12, 15].

### Participants and recruitment

For this current study we only recruited mothers (*n* = 666) who completed the 2-year assessment of the previous trial, including a telephone survey and measurement of child’s height and weight at their homes. Informed consent to this current study was obtained for 662 mothers at the time of their 2-year survey, which became baseline for this current trial. We then randomly allocated the participating mother-child dyads to the intervention group (i.e., receiving combined telephone and SMS intervention) or the control group. The original recruitment criteria of the previous trial included women aged >18 years at 28–34 weeks of pregnancy, were able to communicate in English, had a mobile phone, lived in the recruitment areas, were able to give informed consent and did not have any severe medical conditions.

### Randomization

**Fig. 1:**
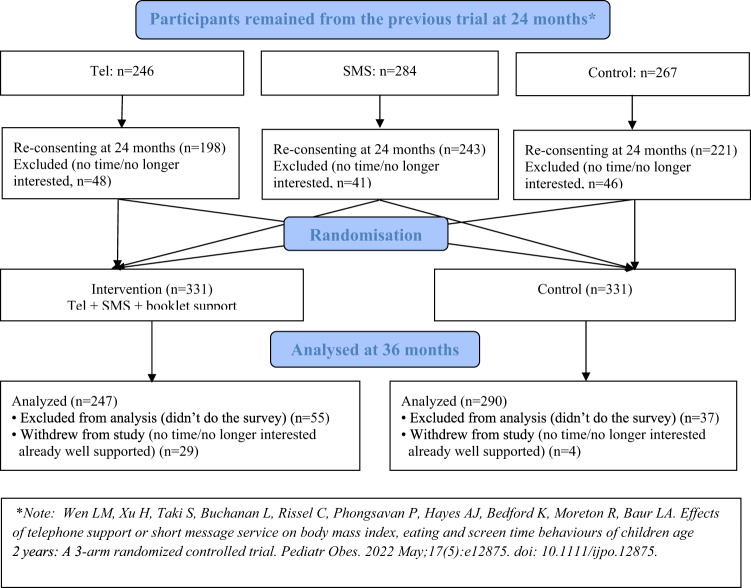
CONSORT diagram. A flowchart of the study participants [12].

We used a stratified randomization method based on participants’ group allocation within the previous trial (see Fig. 1) so that any ‘carry-over’ effect of the previous trial was balanced between the groups. A web-based randomization plan was generated using randomly permuted blocks (*n* = 6) (http://www.randomization.com/).

### Intervention

We developed a 3-stage intervention guided by the Health Belief Model [16], and motivational interviewing techniques as per protocol [17]. The intervention aimed to improve mothers’ parenting behaviors and their own healthy behaviors. Telephone support consisted of protocol-based sessions based on the Australian Dietary Guidelines, early childhood developmental guidelines and the Australian 24-Hour Movement Guidelines for the Early Years (Birth to 5 years) [18]. Each stage of the intervention started with a mailed intervention booklet, followed by a telephone support session and then SMS twice a week for four weeks at three time-points (24–26, 28–30, and 32–34 months of child age). A Child and Family Health Nurse delivered the telephone support session of 30–60 min in duration by going through main intervention messages from the mailed booklets, and then text messages using a 2-way automated SMS system were sent at a predetermined time (10am–1pm) to reinforce the intervention information and key messages in the booklets. A summary of the intervention content be found in the Supplementary Document 1.

### Control

Mothers in the control group received usual care from the local health districts. We also sent out two booklets on information not related to the obesity prevention intervention such as toilet training, language development and sibling relationships as a retention strategy.

### Blinding

A market survey company used a computer-assisted telephone interview (CATI) to collect baseline measures at 2 years and outcome measures at 3 years. The interviewers were blinded to the research hypotheses and treatment allocation. Participating mothers were also blinded to the specific details of the research hypotheses.

### Outcome measurements and data collection

#### Primary outcome

The primary outcome was children’s BMI at 3 years of age. We planned to directly measure weight and height by four research assistants (RAs) via home visits. However, we only managed to measure the height and weight of 30 children (14 intervention, 16 control). Home visiting data collection was stopped due to the COVID-19 pandemic lockdown restrictions in April-October 2020, when most face-to-face health services were suspended. Thus, we had to use the CATI survey to collect children’s height and weight measured by their mothers (*n* = 440) using the measurement kit which was sent out prior to the survey (Supplementary Document 2 about measurements of child height and weight). The measurement kit sourced from a commercial company included the height ruler and detailed instructions for parents on how to measure and record height and weight of their child. We also modified the instructions to suit our study participants. BMI and BMI z-score were calculated using the WHO AnthroPlus v1.0.4.

#### Secondary outcomes

The secondary outcomes at 3 years were BMI z-score, children’s dietary and activity behaviors as reported by their mothers via a telephone survey with a questionnaire. Children’s dietary behavior included vegetable, fruit, fast food and soft drink consumptions as well as feeding practices (i.e., eating in front of the TV and using food for reward). Children’s activity behavior was assessed by their outdoor playtime, screen time and sleep duration. We also collected mothers’ vegetable and fruit consumptions and physical activity and sedentary behaviors. Socio-demographic data were collected by CATI from the previous trial at baseline and then updated at the 2- and 3-year surveys. The questionnaires used for assessing outcomes were the same as those used in the previous Healthy Beginnings Trial [19, 20], and can be found in Supplementary Document 3.

### Sample size

As described in the study protocol [14], we estimated a sample of 506 (253 per group) at age 3 years would allow us to detect a difference of 0.40 kg/m^2^ in mean BMI (SD = 1.60) at the 2-sided 5% significance level with 80% power. This effect size was based on the findings from a 6-month home-based intervention study in the US that detected a decrease in BMI of 0.40 kg/m^2^ with children aged 2–5 years [21].

### Statistical analysis

All statistical analyses were carried out as per study protocol [14] and pre- specified statistical analysis plan and using statistical software STATA 16 (StataCorp 2016). All P-values were two sided and statistical significance was set at the 5% level. Both intention-to-treat analysis with multiple imputations (MI) and complete-case analysis were conducted and reported.

Descriptive analysis was conducted to describe mothers’ demographic characteristics, child BMI and BMI z-score, and secondary outcomes (i.e., children’s dietary behavior and activity behavior). Pearson’s Chi-squared tests examined the differences in mothers’ demographic characteristics between intervention and control groups.

Multiple linear regression models investigated effects of the intervention on child BMI and BMI z-score at 3 years of age. Multiple logistic regression models were fitted to investigate effects of the intervention on secondary outcomes. Adjusted odds ratios (AORs) were calculated. Since the randomization at age 2 years was stratified by group allocation in the previous trial [14], all multiple regression models were adjusted for their previous group allocation. Interactions of intervention allocation with family socio-economic status (SES) (based on annual household income) and language spoken at home were tested. When a significant interaction was found i.e., between intervention allocation and annual household income [note: SES was found to be associated with childhood obesity [4, 22]]; we conducted further subgroup analyses.

Missing data analyses were conducted for study outcomes to examine the patterns and mechanisms of missing data. Little’s test was conducted to test if missing was completely at random (MCAR). Models for missingness were also fitted to examine whether missing was at random. Since the missing was at random, MI by chained equations was used to address potential bias due to missing values. We imputed all missing values for the full intention to treat analysis of all 662 randomized participants. The imputation model predicting missing outcome values was based on all plausible observed values of outcomes, dietary and activity behaviors and family demographics at baseline (2 years of age) and at 3 years of age. We used 20 imputations each time which gave a relative efficiency of 99% [23], a similar approach to that used in our previous studies [11, 12, 19, 20].

## Results

### Characteristics of study participants and follow-up

Figure 1 shows 662 mothers from the previous trial completed the 2-year survey (i.e., baseline of this study). Table 1 shows similar distributions of mothers’ demographic characteristics and child BMI and BMI z-score except for the proportion of overweigh and obesity at baseline (at 2 years) between the two groups. There were 257 (39%) participants from families with annual household incomes less than AUD$80K (note: Sydney median household income was AUD$109K in 2020). At 3 years, of 662 participating mother-child dyads, 537 (81%) mothers (247 intervention; 290 control) completed the telephone survey and 470 (71%) children had their height and weight measured: 30 (6%) measured by RAs and 440 (94%) measured and reported by mothers. There were no significant differences in mothers’ demographic characteristics between those who completed and did not complete the 3-year survey, except language spoken at home (Supplementary Table 1). More mothers were excluded from the analysis in the intervention than the control (Fig. 1).

**Table 1:** Families’ characteristics, child BMI and BMI z-score by group allocation at 2 years (baseline).

Variables	Total *N* = 662 *n* (%)	Intervention *n* = 331 *n* (%)	Control *n* = 331 *n* (%)
Mother’s age			
<30 years	183 (28)	101 (31)	82 (25)
≥30 years	479 (72)	230 (69)	249 (75)
Country of birth			
Australia	251 (38)	124 (37)	127 (38)
Other	411 (62)	207 (63)	204 (62)
Language spoken at home			
English	351 (53)	179 (54)	172 (52)
Other	311 (47)	152 (46)	159 (48)
Annual household income			
<$ 80.000	255 (39)	123 (37)	132 (40)
≥$ 80,000	407 (61)	208 (63)	199 (60)
Employment status			
Employed	444 (67)	223 (67)	221 (67)
Other	218 (33)	108 (33)	110 (33)
Marital status			
Married/de-facto partner	629 (95)	317 (96)	312 (94)
Other	33 (5)	14 (4)	19 (6)
Education level			
Up to HSC to TAFE/Diploma	201 (30)	108 (33)	93 (28)
University	461 (70)	223 (67)	238 (72)
Father’s employment status			
Employed	606 (91)	305 (92)	301 (91)
Other	56 (9)	26 (8)	30 (9)
Father’s education level			
Up to HSC to TAFE/Diploma	247 (38)	115 (35)	132 (40)
University	415 (62)	216 (65)	199 (60)
Child sex			
Boy	333 (50)	173 (52)	160 (48)
Girl	329 (50)	158 (48)	171 (52)
Overweight and Obesity^a^	125 (19)	49 (15)	76 (23)
	**Mean (SD)**	**Mean (SD)**	**Mean (SD)**
Child BMI	16.91 (1.52)	16.83 (1.44)	17.00 (1.59)
Child BMI z-score	0.85 (1.03)	0.79 (0.98)	0.91 (1.07)

*HSC* Higher School Certificate (Year 12), *TAFE* Technical and Further Education.

^a^Using the International Obesity Task Force BMI cut offs for young children.

### Comparisons of the primary outcome between the groups

As shown in Table 2, there was no statistically significant difference in BMI or BMI z-score between children in the intervention group and the control group. Since the test of interaction between annual household income and intervention allocation in the complete-case analysis was significant (*P* = 0.049), we conducted subgroup analysis by annual household income for both complete-case analysis and MI analysis. In MI analysis, among children from a lower income family (annual household income <AUD$80,000), the intervention group had significant lower BMI than the control (mean difference −0.59 kg/m^2^, 95% CI −1.15 to −0.03, *P* = 0.040) at 3 years of age. In complete-case analysis, among children from a lower income family, the intervention group had significant lower BMI (mean difference −0.74 kg/m^2^, 95% CI −1.39 to −0.08, *P* = 0.028) and lower BMI z-score (mean difference −0.51, 95% CI −0.98 to −0.05, *P* = 0.032) than the control group.

**Table 2:** Comparisons of the primary outcome (BMI and BMI z-score) between the intervention and control groups at 3 years of age.

Primary outcomes	Intervention Mean (SD)	Control Mean (SD)	Intervention - Control Mean difference (95% CI)^a^	*P*
Complete-case analysis *n* = 470	*n* = 211	*n* = 259		
BMI	16.40 (1.70)	16.67 (1.97)	−0.27 (−0.61 to 0.06)	0.111
BMI z-score	0.59 (1.25)	0.77 (1.38)	−0.18 (−0.42 to 0.06)	0.148
Subgroup analysis *n* = 437				
Annual household income < $ 80,000 *n* = 149	*n* = 66	*n* = 83		
BMI	16.21 (1.81)	16.95 (2.23)	−0.74 (−1.39 to −0.08)	0.028
BMI z-score	0.43 (1.35)	0.94 (1.56)	−0.51 (−0.98 to −0.05)	0.032
Annual household income ≥ $ 80,000 *n* = 288	*n* = 135	*n* = 153		
BMI	16.52 (1.61)	16.53 (1.72)	−0.01 (−0.40 to 0.38)	0.954
BMI z-score	0.70 (1.16)	0.69 (1.24)	0.01 (−0.27 to 0.29)	0.926
Multiple imputation *n* = 662	*n* = 331	*n* = 331		
BMI	16.38 (2.14)	16.68 (2.07)	0.30 (−0.61 to 0.02)	0.066
BMI z-score	0.58 (1.52)	0.77 (1.47)	0.19 (−0.41 to 0.03)	0.096
Subgroup analysis *n* = 662				
Annual household income < $ 80,000 *n* = 255	*n* = 123	*n* = 132		
BMI	16.26 (2.22)	16.84 (2.37)	−0.59 (−1.15 to −0.03)	0.040
BMI z-score	0.46 (1.63)	0.86 (1.65)	−0.41 (−0.81 to 0.01)	0.048
Annual household income ≥ $ 80,000 *n* = 407	*n* = 208	*n* = 199		
BMI	16.45 (2.18)	16.56 (1.92)	−0.11 (−0.51 to 0.28)	0.572
BMI z-score	0.65 (1.56)	0.71 (1.36)	−0.06 (−0.34 to 0.23)	0.691

^a^Mean differences from multiple linear regression models adjusted previous intervention allocation.

### Comparisons of secondary outcomes between the groups

Based on MI analysis, Table 3 shows statistically significant differences were observed between the groups i.e., according to mothers’ self-reports, children in the intervention group were more likely not to eat in front of the TV [AOR 1.79 (95% CI 1.17–2.73) *P* = 0.008], more likely to meet the 6 intervention recommendations for dietary behavior [AOR 1.73 (95% CI 0.99–3.02) *P* = 0.054] and meet 3 activity intervention recommendations for activity behavior [AOR 1.72 (95% CI 1.11–2.67) *P* = 0.015] than those in the control group respectively. These results were similar to those from the complete-case analysis as shown in Supplementary Table 2.

**Table 3:** Comparisons of secondary outcomes of children and mothers between intervention and control groups at 3 years of age (intention-to-treat analysis with multiple imputations).

Secondary outcomes	Intervention Total = 331 *n* (%)	Control Total = 331 *n* (%)	Intervention vs. Control AOR (95% CI)
Children			
Fruit consumption			
≥1 serves/day	317 (96)	314 (95)	1.26 (0.45–3.55)
Vegetable consumption			
≥2.5 serves/day	119 (36)	97 (29)	1.36 (0.95–1.95)
Fast food			
No	122 (37)	108 (33)	1.21 (0.85–1.72)
Soft drink			
No	270 (82)	274 (83)	0.92 (0.59–1.44)
Eating in front of TV			
No	261 (79)	224 (68)	1.79 (1.17–2.73) *P* = 0.008
Food for reward			
No	267 (81)	261 (79)	1.12 (0.71–1.76)
*Dietary behavior*			
*Meeting all 6 recommendations above*^a^	40 (12)	24 (7)	1.73 (0.99–3.02) *P* = 0.054
Outdoor play time			
≥2 hours/day	229 (69)	207 (63)	1.36 (0.96–1.91)
Screen time			
<1 hour/day	116 (35)	102 (31)	1.22 (0.85–1.76)
Daily sleep duration			
≥11 hours/day	242 (73)	219 (66)	1.39 (0.97–2.00)
*Physical activity/screen/sleep behavior*			
*Meeting all 3 recommendations above*^a^	75 (23)	48 (14)	1.72 (1.11–2.67) *P* = 0.015
Mothers			
Fruit consumption			
≥2 serves/day	167 (51)	183 (55)	0.82 (0.60–1.14)
Vegetable consumption			
≥5 serves/day	48 (14)	36 (11)	1.38 (0.82–2.34)
Physical activity time			
>150 minutes/week	241 (73)	254 (77)	0.82 (0.55–1.22)
Sedentary time			
≤4 hours/day	138 (42)	143 (43)	1.07 (0.76–1.51)

*AOR* adjusted odds ratio, adjusted for previous intervention allocation.

^a^Based on Australian Institute of Health and Welfare 2020. Australia’s children. Cat. no. CWS 69. Canberra: AIHW

### Process indicators

Between ages 2 and 3 years, of 331 mothers allocated to the intervention group, 281 (85%) mothers completed Stage 1 telephone support session, 250 (76%) completed Stage 2 telephone session, and 210 (63%) completed Stage 3 telephone session. Further, 183 (55%) mothers completed all 3 sessions, 72 (22%) completed 2 sessions, 48 (15%) received 1 session, and 28 (8%) mothers did not complete any session. We were unable to determine the number of mothers who did not receive any SMS support.

## Discussion

### Principal findings of the study

This is the first RCT to investigate the effect of a staged, nurse-led telephone plus SMS support intervention on children’s BMI at age 3 years. Our findings suggest that although the combined intervention had no significant overall effect on BMI, the intervention was significantly associated with lower mean BMI of children from a lower household income family. The intervention was also significantly associated with reduced odds of eating in front of the TV and improved odds of meeting the dietary and activity intervention recommendations among the intervention participants.

### Meaning of the study

This study was conducted during March 2019 and October 2020 under extraordinary circumstances where the COVID-19 pandemic outbreak and associated lockdown measures in NSW took place. According to the CONSERVE 2021 Statement [24] we had to modify the study protocol including the intervention content covering COVID-19 related information and used mailed measurement kits for parents to take measures of height and weight instead of this being undertaken directly by research assistants during a home visit. It is possible that the lack of overall intervention effect on BMI could be associated with the modifications to the study protocol and also changes in participants’ behaviors and life priorities caused by the pandemic. However, the finding was not surprising given the limited quality evidence available [25, 26]. In a 2022 systematic review of prevention and treatment of childhood overweight and obesity in children up to 5 years old [26], the authors suggested there was a differential effect of interventions on measures of childhood obesity by setting, with interventions conducted in a home setting being more effective than eHealth coaching.

Our findings from subgroup analyses support some limited evidence from previous studies that childhood obesity prevention interventions may be more effective for children from lower socio-economic families and communities [27]. Existing studies show children from lower socio-economic families and communities are at higher risk of overweight and obesity [4, 5, 28, 29]; arguably, obesity prevention programs should be targeting these socioeconomically disadvantaged families.

The intervention effect on children’s eating in front of the TV was important and relevant to childhood obesity prevention. A systematic review found that eating while watching TV is associated with poorer diet quality among children, with more frequent consumption of sugar-sweetened beverages and high-fat, high-sugar foods and fewer fruits and vegetables [30]. A Canadian study also suggested that eating while watching television leads to increased energy intake by delaying normal mealtime satiation and reducing satiety signals from previously consumed foods [31]. The intervention effects on improving children’s meeting the dietary and activity intervention recommendations were also encouraging, which could contribute partly to BMI reduction among children receiving the intervention.

### What the study adds

First, the study provides timely evidence on a nurse-led staged telephone support intervention with SMS for preventing childhood obesity risk of toddlers. In particular, among low-income families there has been an increasing use of telephone or SMS for health service provision during the COVID-19 pandemic. The reduced mean BMI of children from lower income families is of public health significance in decreasing obesity prevalence at the population level [19, 32]. Second, given scarce public health prevention funding available, both SMS and telephone interventions would be more affordable than face-to-face (i.e., home visiting) interventions, with a greater potential to be scaled up. Third, the study was conducted partly during the pandemic when vulnerable families were very much in need of health service support as most face-to-face services were suspended, while telehealth intervention could fill in the service gaps and yield additional co-benefits such as mental health support [33].

### Unanswered questions and future research

In our study the intervention was effective in improving dietary and activity behaviors based on mothers’ self-reported estimates, but reducing BMI of children was only found among lower household income families. A possible explanation could be that the intervention may have missed other important risk factors that contribute to childhood obesity. It is therefore important for future research to explore the development of comprehensive interventions for assessing childhood obesity. In addition, with limited studies available on cost-effectiveness of interventions, future research should examine the cost-effectiveness of various intervention approaches. In this study we conducted three staged telephone and SMS interventions to mothers of children aged 2 years. The impact of the intervention dose (e.g., frequency of telephone calls or text messages) is also worth investigation. In addition, the impact of the pandemic on children’s eating, physical activity and screen time behaviors, as well as the intervention effects, requires further investigation as the pandemic significantly impacted the mental health of mothers with young children and their means of communication with health professionals [34].

### Strengths and limitations

Strengths included the use of an RCT to test an evidence-informed intervention. We published the study protocol [14] with a pre-specified statistical analysis plan prior to its commencement. This trial was built directly on a previous 3-arm RCT [11–13] to address a known gap in obesity prevention for children aged 2–3 years. In this way we used existing research infrastructure and systems (i.e., telephone or SMS support) in place to run the study and optimized the use of an already engaged population of study participants. To minimize the ‘carried over effect’ (i.e., exposure to the previous study), we used a stratified randomization by previous group allocation, and took into account previous group allocation of the previous study in data analyses. We also used well-developed survey questionnaires that were widely used in the past studies [11, 19, 20] to assess the intervention outcomes.

However, the generalizability of this study may be limited due to the use of existing study participants who could be highly committed to a public health research program. We were also unable to carry out weight and height measures objectively by RAs through home visits as planned due to the COVID-19 pandemic. The use of mail-out of measurement tools for parents to measure was an alternative option under extenuating circumstances. It is likely that the measurement errors would be balanced out by the RCT design. The complete-case analysis for BMI may be under-powered to detect the intervention effect as we did not reach the required sample size (*n* = 506). The intervention participants’ ‘fatigue’ was observed as we found that more participants from the intervention (*n* = 84) were lost to follow-up than the control (*n* = 41). However, no significant differences were found in overall characteristics of those remaining in the study and lost to follow-up. In addition, the redeployment of intervention nurses for COVID-19 responses resulted in their limited availability to deliver the program as scheduled. Further, we are fully aware that some limitations are associated with subgroup analysis, such as false positives due to multiple comparisons, or false negatives due to inadequate power [35]. We decided to test an interaction between intervention and household income (a proxy for family SES), which was specified a priori since the association of SES with childhood obesity is well established. The household income of participants was collected prior to their randomization and the hypothesis and direction of the subgroup effect was pre-specified. We only conducted subgroup analyses for the primary outcome. Thus, our subgroup analysis met most criteria for credible subgroup effects [35, 36]. However, caution needs to be taken when interpreting the results of subgroup analyses. The finding of a positive effect on lower income group could occur by chance although we had pre-specified the hypothesis tests for this sub-group analysis in our statistical analysis plan rather than post hoc testing, and only conducted sub-group analyses when an interaction was found. However, our findings could be considered potentially hypothesis generating, warranting further investigation on whether an early obesity prevention intervention targeting low SES population has an optimal effect on child BMI.

## Conclusion

This study concluded that a staged, combined telephone and SMS support to mothers of children aged 2 years was associated with reduced children’s BMI at age 3 years only among those families with a low household income. The intervention was also associated with reduced odds of eating in front of the TV as well as with improved dietary and activity behaviors based on mothers’ self-reported estimates. Telephone and SMS based support targeted at low SES families could be a pertinent strategy to reduce current inequalities in childhood obesity.

## Supplementary information

Supplementary Document 1

Supplementary document 2

Supplementary document 3

Supplementary Table 1

Supplementary Table 2

## Data Availability

De-identified data and material can be available on request pending ethics approval.
