# Supplementary material for: Twelve-month effectiveness of telephone and SMS support to mothers with children aged 2 years in reducing children’s BMI: a randomized controlled trial
Source: Int J Obes (Lond). 2023 Apr 22;47(9):791–8. doi: 10.1038/s41366-023-01311-7 (PMC10121422; doi:10.1038/s41366-023-01311-7)
Supplement: Supplementary file 1 — Supplementary Document 1 [file 41366_2023_1311_MOESM1_ESM.docx]

**Supplementary Document 1:** **A summary of the intervention content**

| **Component/focus** | **Main contents** | **Sample of SMS** |
| --- | --- | --- |
| Repeated exposure to healthy foods, limiting exposure to non-core foods | Recommended dietary intake of food groups, food label reading, healthy food environments, eating family meals, managing external influences on dietary intake | “Messy eating and playing with food are normal parts of [baby’s name] development when learning to eat independently. Check out the Healthy Beginnings booklet for tips to help with feeding” |
| Promotion of choosing water as a drink from a cup and exclude sugar sweetened beverages | Encourage drinking healthy drinks from a cup and encouraged to stop offering milk in a bottle oral health | “Drinking water from a cup is the only drink that [baby’s name] needs. Fruit juice, soft drinks, flavoured milk can cause teeth decay” |
| Promotion of responsive complementary feeding strategies | Hunger and satiety cues, strategies to manage fussy eating behaviours | “At 2 - 3 years it is normal for toddlers’ appetite to go up and down. Keep on providing a variety of healthy foods and it is up to [baby’s name] to decide how much food to eat.” |
| Promotion of incorporating physical activity into children’s daily routine | Encourage active and quiet play, support the development of Fundamental Movement Skills, developmental milestones, child safety | “It is recommended that toddlers should be active every day for at least 3 hours throughout the day. Playing in the park, kicking a ball or catching & throwing are some fun activities” |
| Advised limited (less than 1 hour/day) screen-time (TV, DVDs, computers, smartphones, iPads/tablets) | Limit screen time, encourage interactive, quiet (reading, singing, music, drawing, dress-ups) and active (dancing, playground, running, walking) play | “Your baby needs to be active every day and does not need any screen time.  Playing with other children in a safe, supervised area will help them develop socially.” |
| Promotion of fostering healthy sleeping habits | Developing a sleep schedule, developing a bedtime routine, understanding sleep patterns, address common challenges affecting sleep behaviours | “At 2 - 3 years toddlers need to sleep between 12 – 13 hours a day. The Healthy Beginnings booklets have tips on how to develop a healthy sleep routine. For more support send us a text |
| Key Features of the intervention delivery mode:   - Key messages were delivered at three stages: 24 – 26, 28 – 30 and 32– 34 months - A Healthy Beginnings booklet* was sent to participants, which provides information on relevant topics for each stage - Two SMS messages were sent per week tailored to the child’s development stages. - Telephone calls using Anticipatory Guidance to deliver the content at each milestone by trained Child and Family Health Nurses | | |

* can be found at <http://www.healthybeginnings.net.au/resources-1-1>
