# Supplementary material for: Twelve-month effectiveness of telephone and SMS support to mothers with children aged 2 years in reducing children’s BMI: a randomized controlled trial
Source: Int J Obes (Lond). 2023 Apr 22;47(9):791–8. doi: 10.1038/s41366-023-01311-7 (PMC10121422; doi:10.1038/s41366-023-01311-7)
Supplement: Supplementary file 2 — Supplementary document 2 [file 41366_2023_1311_MOESM2_ESM.docx]

**Supplementary document 2: Measurements of child height and weight**

**Invitation Letter**

Due to the COVID interruption, objectively weight and height measurement was not possible during the data collection in 2020. As a result, participants were sent an invitation letter together with a measurement kit four weeks before the child turned 4 years old. Participants were encouraged to measure their child’s weight and height and texted the weight and height data to the Healthy Beginnings Team. The measurement kids included an instruction sheet and a height ruler.

**
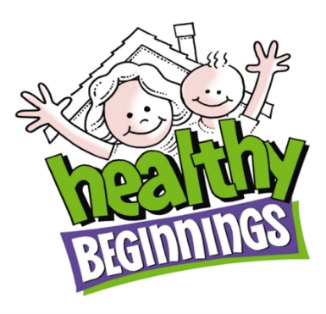
**

«AddressBlock»

«GreetingLine»


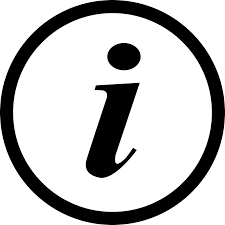
Hope you and family are well. Thank you for your continuing participation in the Healthy Beginnings Program. «child_first_name»’s weight and height data at 4 years old are important indicators of «child_first_name»’s growth and development. In this mail-out, we have provided you with a measurement kit that includes:

- An instruction sheet to measure and record «child_first_name»’s height and weight
- A height ruler to measure «child_first_name»’s height
- A Healthy Beginnings lunchbox as a thank you gift


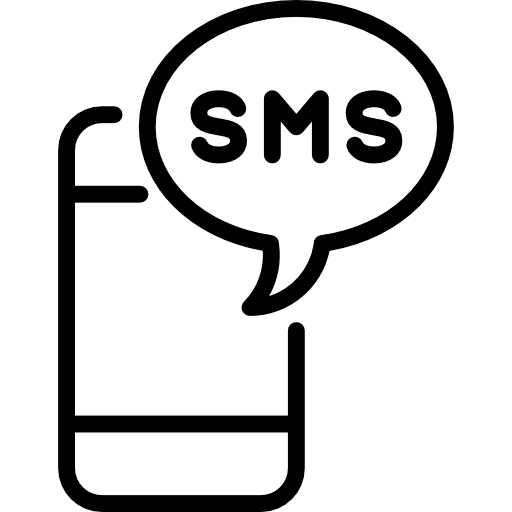
After the measurement, can you **please text us 0457 111 555 «child_first_name»’s and your weight and height data, and measurement date?** Or email us at [SLHD-HealthyBeginnings@health.nsw.gov.au](mailto:SLHD-HealthyBeginnings@health.nsw.gov.au)

[
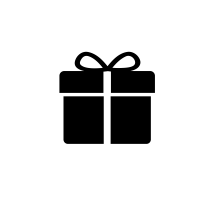
](https://www.google.com.au/url?sa=i&rct=j&q=&esrc=s&source=images&cd=&cad=rja&uact=8&ved=0ahUKEwjDsvfToK3TAhXDsJQKHc2CDdIQjRwIBw&url=https://thenounproject.com/term/gift/105033&psig=AFQjCNHmD8885QKkaz-oZAt9hZ78jxYT1Q&ust=1492579106346637)

By providing us with the self-measured weight and height data. You will be eligible to **enter a Healthy Beginnings lucky draw of $100 shopping voucher**.

Also a reminder that «child_first_name» is due for 3 year Developmental (Bluebook) Check, and 4 year immunisation. You can also visit your local Child and Family Health Centre or GP to measure «child_first_name»’s weight and height.

If at any time your circumstances change or you have any questions or concerns, please contact us via email at [SLHD-HealthyBeginnings@health.nsw.gov.au](mailto:SLHD-HealthyBeginnings@health.nsw.gov.au)

or by texting 0457 111 555 or calling 0436 924 200.

With regards,

The Healthy Beginnings Study Project Team

**Measurement Kit**

Weight and health measurement kit sourced from a commercial company Better Health Co. (betterhealthcompany.org) was mailed out to the participants. This measurement kit was used by the NSW Statewide Go4Fun Program. We modified the instructions to suit our study participants. This measurement is for reviewers’ information only, not for distributions to the readers.


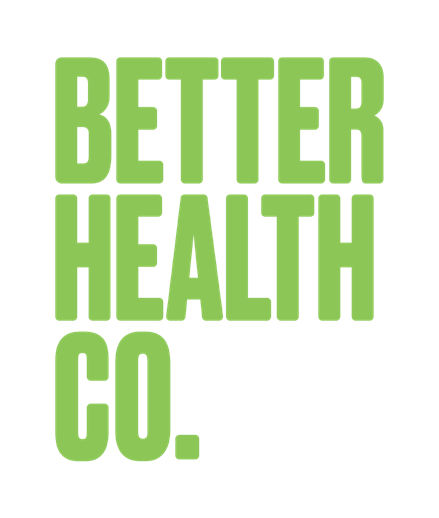


**Height**

# Measurements instructions

Use the height ruler you received in this pack to measure your child’s height.

1.
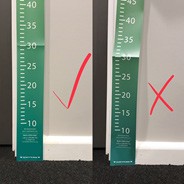
Find a wall or door frame with no skirting boards and attach the ruler with a small amount of blue tac or sticky tape.
2. Make sure that the bottom of the ruler is level with the floor and that the tape is straight.
3. Follow the instructions to fold together the height measuring triangle.
4. Ensure your child has removed their shoes, any heavy outer garments and hair accessories or hats.
5. Ask your child to stand with their back to the ruler, stand up straight and look directly ahead. Remind your child to breathe normally.
6. Once your child is in position, place the triangle lightly on top of their head. Take the measurement from where it says ‘read height here’ on the height triangle.
7. Record your child’s height on the attached measurement form in centimetres


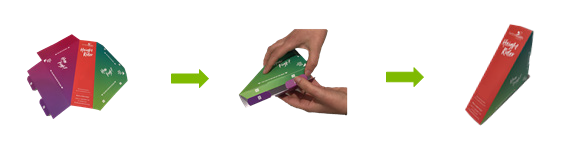

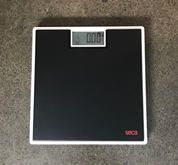


**Weight**

1. Place scales on a hard, level surface (e.g. floorboards or tiles). Make sure that the scales read 0.0kg before any weight is placed on them.
2.
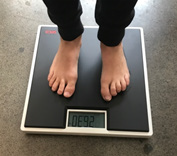
Ask your child to remove shoes, heavy outer clothing and empty pockets.
3. Ask your child to stand centred on the scale, look straight ahead (rather than down at the scale), and stay still. Remind child to breathe normally.
4. Record their weight on the attached measurement form in kilograms to one decimal place.


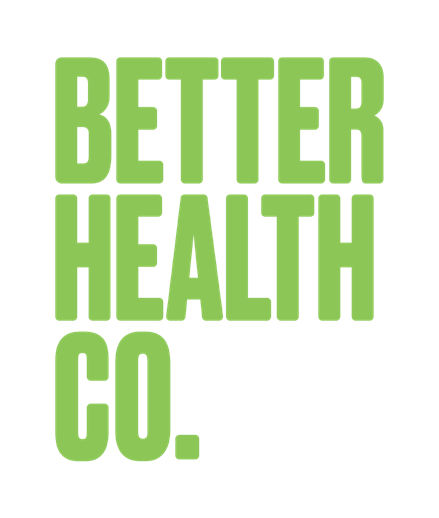


# Measurements form

Please text the weight and height measurements of yours and your child to Healthy Beginnings Team 0457 111 555.

Your child is due for their 4 year developmental (bluebook) check, and 4 yer immunisation. You can also visit your local Child and Family Health Centre or GP to measure your child’s weight and height.

**Child’s details**

| Child’s name |  | | | | | | | | |
| --- | --- | --- | --- | --- | --- | --- | --- | --- | --- |
| Measurement date (dd/mm/yyyy) |  |  | / / | |  |  |  |  |  |
| Height (cm) |  |  |  | cm | |  |  |  | |
| Weight (kg) |  |  |  | . kg | |  |  |  | |
| **Parent / carer’s details** | |  |  | | |  |  |  | |
| Parent / carer’s name |  |  |  | | |  |  |  | |
| Measurement date (dd/mm/yyyy) |  |  | / / | |  |  |  |  |  |
| Height (cm) |  |  |  | cm | | | | | |
| Weight (kg) |  |  |  | . kg | | | | | |
