# Supplementary material for: Twelve-month effectiveness of telephone and SMS support to mothers with children aged 2 years in reducing children’s BMI: a randomized controlled trial
Source: Int J Obes (Lond). 2023 Apr 22;47(9):791–8. doi: 10.1038/s41366-023-01311-7 (PMC10121422; doi:10.1038/s41366-023-01311-7)
Supplement: Supplementary file 3 — Supplementary document 3 [file 41366_2023_1311_MOESM3_ESM.docx]

**Supplementary document 3: Secondary outcomes and assessment questions**

| **Secondary Outcomes** | **Assessment Questions** |
| --- | --- |
| **Children** |  |
| **Fruit consumption**  <1 serves/day  ≥1 serves/day | - How many serves of fruit does your child usually eat in a day? (1 serve = 1 medium piece or two small pieces of fruit or 1 cup of diced pieces)    ______serves per day   ______serves per week   Doesn’t eat fruit   Don’t know [don’t read]   Refused [don’t read] |
| **Vegetable consumption**  <2.5 serves/day  ≥2.5 serves/day | - How many serves of vegetables does your child usually eat in a day? (1 serve = ½ cup cooked vegetables or 1 cup of salad vegetables)    ______serves per day   ______serves per week   Doesn’t eat vegetables   Don’t know [don’t read]   Refused [don’t read] |
| **Fast food consumption**  Yes  No | - How often does your child have meals or snacks such as burgers, pizza, chicken or chips from places like McDonalds, Hungry Jacks, Pizza Hut, KFC, Red Rooster or local takeaway places?    ______times per day   ______times per week   ______times per month   Rarely/never   Don’t know [don’t read]   Refused [don’t read] |
| **Soft drink consumption**  Yes  No | - How many cups of **soft drink** (such as lemonade), **cordial** or **sports drinks** (such as Gatorade) does your child usually drink? (1 cup = 250ml, 1 can soft drink = 1 ½ cups, one bottle Gatorade = 2 cups) Do not include diet drinks.    ______times per day   ______times per week   ______times per month   Doesn’t drink soft drink or cordial   Don’t know [don’t read]   Refused [don’t read] |
| **Feeding practices** | - How often does [child’s name] |
| **Eat in front of the TV**  Yes  No |  Often   Sometimes   Very occasional   Not at all   Don’t know [don’t read] |
| **Being rewarded with food to encourage good behaviour**  **(Food for reward)**  Yes  No |  Often   Sometimes   Very occasional   Not at all   Don’t know [don’t read] |
| **Dietary behaviour**  Doesn’t meet all 6 recommendations  Meet all 6 recommendations | Based on fruit (≥1 serve/day), vegetable (≥2.5 serve/day), fast food (No), soft drink (No) consumption, eat in front of the TV (No) and food reward (No). |
| **Outdoor play time**  >2 hours/day  ≤2 hours/day  **Calculation:** | - Think for a moment about a typical weekday for your child in the last month. How much time would you say your child spends playing outdoors on a typical weekday?    _______minutes _______hours   Don’t know [don’t read]   Refused [don’t read]   - Now think about a typical weekend day for your child in the last month. How much time would you say your child spends playing outdoors on a typical weekend day?    _______minutes _______hours   Don’t know [don’t read]   Refused [don’t read]  **Outdoor play time=(time on weekday × 5 days + time on weekend day × 2 days)/7 days** |
| **Screen time** | - During a usual week, how much time does your child spend doing each of the following at home? |
| Watching television programs (include watching Netflix, Foxtel, or YouTube) | Total time Monday to Friday   _______minutes _______hours  Total time on weekends   _______minutes _______hours |
| Watching smart phone, iPad, Tablet, etc. | Total time Monday to Friday   _______minutes _______hours  Total time on weekends   _______minutes _______hours |
| Using a computer or laptop | Total time Monday to Friday   _______minutes _______hours  Total time on weekends   _______minutes _______hours |
| Playing with an electronic game system (e.g. Playstation, Xbox, PSP etc.) | Total time Monday to Friday   _______minutes _______hours  Total time on weekends   _______minutes _______hours |
| **Calculation** | **Screen time=(time on weekdays + time on weekends)/7** |
| **Sleep** |  |
| **Sleep duration**  <11 hours/day  ≥11 hours/day  **Calculation** | - What time does your child usually go to bed at night?    __________ pm   Don’t know [*don’t read*]   Refused [*don’t read*]   - What time does your child usually fall asleep at night?    __________ pm   Don’t know [*don’t read*]   Refused [*don’t read*]   - How many times does your child usually wake during the night?   _________ times  None  Don’t know [don’t read]  Refused [don’t read]   - For how long does he/she usually stay awake?    ______ minutes ______ hour(s)   Don’t know [don’t read]   Refused [don’t read]   - What time does your child usually wake up in the morning to start the day?    __________ am   Don’t know [*don’t read*]   Refused [*don’t read*]   - During the day, how many sleeps does your child usually have?    _________ sleeps   None   Don’t know [don’t read]   Refused [don’t read]   - At what time of day and for how long does he/she usually sleep at these times?  \|  \| Approx. time of day \| Approx. length of sleep \| \| --- \| --- \| --- \| \| 1^st^ daytime sleep \| am/pm \| <30mins ½-1hr 2-3hrs >3hrs \| \| 2^nd^ daytime sleep \| am/pm \| <30mins ½-1hr 2-3hrs >3hrs \| \| 3^rd^ daytime sleep \| am/pm \| <30mins ½-1hr 2-3hrs >3hrs \|   Daily sleep duration=nocturnal sleep time +nap time during  the day |
| **Activity behaviour**  Doesn’t meet all 3 recommendations  Meet all 3 recommendations | Based on outdoor play time (≥2 hrs/day), screen time (<1 hr/day) and daily sleep duration (≥11 hrs/day). |
| **Mothers** |  |
| **Fruit consumption**  <2 serves/day  ≥2 serves/day | - How many serves of fruit do you usually eat each day? (Include fresh, dried, frozen and tinned fruit. One serve = 1 medium piece or 2 small pieces of fruit or 1 cup of diced pieces)    ______serves per day   ______serves per week   Don’t eat vegetables   Don’t know [don’t read]   Refused [don’t read] |
| **Vegetable consumption**  <5 serves/day  ≥5 serves/day | - How many serves of vegetables do you usually eat each day? (Include fresh, dried, frozen and tinned vegetables. One serve = ½ cup cooked or 1 cup of salad vegetables)    ______serves per day   ______serves per week   Don’t eat vegetables   Don’t know [don’t read]   Refused [don’t read] |
| **Physical activity** |  |
| **Vigorous activity**  **Calculation** | - During the last 7 days, on how many days did you do vigorous physical activities like heavy lifting, digging, aerobics, or fast bicycling?    ________# days   No vigorous physical activities   Don’t know [don’t read]   Refused [don’t read]   - How much time did you usually spend doing vigorous physical activities on one of those days?    _______minutes per day  _______hours per day   Don’t know/Not sure   Refused [don’t read]  Vigorous PA time per week=number of days × time/day |
| Moderate activity  **Calculation** | - During the last 7 days, on how many days did you do moderate physical activities like carrying light loads, bicycling at a regular pace, or doubles tennis? Do not include walking.    ________# days   No moderate physical activities   Don’t know [don’t read]   Refused [don’t read]   - How much time did you usually spend doing moderate physical activities on one of those days?    _______minutes per day  _______hours per day   Don’t know/Not sure   Refused [don’t read]  Moderate PA time per week=number of days × time/day |
| **Walk** | - During the last 7 days, on how many days did you walk for at least 10 minutes at a time?    ________# days   No walking   Don’t know [don’t read]   Refused [don’t read]   - How much time did you usually spend walking on one of those days?    _______minutes per day  _______hours per day   Don’t know/Not sure   Refused [don’t read]  Walk time per week=number of days × time/day |
| **PA time Calculation**  <150 minutes/week  ≥150 minutes/week | PA time = Vigorous PA time × 2 + Moderate PA time + Walk time |
| **Sedentary time**  ≤4 hours/day  >4 hours/day | - During the last 7 days, how much time did you spend sitting on a week day?    _____ hours per day   _____ hours per week   Don’t know/Not sure [don’t read]   Refused [don’t read] |
