# Supplementary material for: Twelve-month effectiveness of telephone and SMS support to mothers with children aged 2 years in reducing children’s BMI: a randomized controlled trial
Source: Int J Obes (Lond). 2023 Apr 22;47(9):791–8. doi: 10.1038/s41366-023-01311-7 (PMC10121422; doi:10.1038/s41366-023-01311-7)
Supplement: Supplementary file 4 — Supplementary Table 1 [file 41366_2023_1311_MOESM4_ESM.docx]

**Supplementary Table 1. Families’ baseline characteristics by survey completion at 3 years**

| **Mother’s baseline demographics** | **3 year survey completion** | | |
| --- | --- | --- | --- |
|  | **Yes**  **n (%)** | **No**  **n (%)** | **P** |
| **Age (years)** |  |  | 0.607 |
| 16-24 | 34 (7) | 9 (7) |  |
| 25-29 | 109 (20) | 31 (25) |  |
| 30-34 | 211 (39) | 51 (41) |  |
| 35-39 | 146 (27) | 28 (22) |  |
| 40-49 | 37 (7) | 6 (5) |  |
| **Country of birth** |  |  | 0.130 |
| Australia | 211 (39) | 40 (32) |  |
| Overseas | 326 (61) | 85 (68) |  |
| **Language spoken at home** |  |  | 0.025 |
| English | 296 (55) | 55 (44) |  |
| Other | 241 (45) | 70 (56) |  |
| **Annual household income** |  |  | 0.413 |
| <$ 40,000 | 59 (11) | 10 (8) |  |
| $ 40,000 to $79,999 | 116 (22) | 31 (25) |  |
| ≥$ 80,000 | 316 (59) | 69 (55) |  |
| Don’t know | 46 (8) | 15 (12) |  |
| **Employment status** |  |  | 0.307 |
| Employed | 365 (68) | 79 (63) |  |
| Other | 172 (32) | 46 (37) |  |
| **Marital status** |  |  | 0.572 |
| Married/de-facto partner | 508 (95) | 120 (96) |  |
| Other | 28 (5) | 5 (4) |  |
| **Education level** |  |  | 0.085 |
| University | 381 (71) | 79 (63) |  |
| Up to HSC*/TAFE^ | 155 (29) | 46 (37) |  |
| **Father’s education level** |  |  | 0.679 |
| University | 326 (63) | 72 (61) |  |
| Up to HSC/TAFE | 191 (37) | 46 (39) |  |
| **Father’s employment status** |  |  | 0.372 |
| Employed | 475 (91) | 115 (93) |  |
| Other | 47 (9) | 8 (7) |  |
| **Child sex** |  |  | 0.107 |
| Boy | 262 (49) | 71 (57) |  |
| Girl | 275 (51) | 54 (43) |  |

Note:

Sample size is not necessarily 662 due to missing values.

P: P values of Pearson’s Chi-squared tests.

*HSC: Higher School Certificate (Year 12), ^TAFE: Technical and Further Education
