# Supplementary material for: Twelve-month effectiveness of telephone and SMS support to mothers with children aged 2 years in reducing children’s BMI: a randomized controlled trial
Source: Int J Obes (Lond). 2023 Apr 22;47(9):791–8. doi: 10.1038/s41366-023-01311-7 (PMC10121422; doi:10.1038/s41366-023-01311-7)
Supplement: Supplementary file 5 — Supplementary Table 2 [file 41366_2023_1311_MOESM5_ESM.docx]

**Supplementary Table 2. Comparisons of secondary outcomes of children and mothers between intervention group and control group at 3 years of age (complete-case analysis)**

| **Secondary outcomes** | **Intervention**  **Total=331**  **n (%)** | **Control**  **Total=331**  **n (%)** | **Intervention vs. Control**  **AOR (95% CI)** |
| --- | --- | --- | --- |
| **Children** |  |  |  |
| **Fruit** |  |  |  |
| ≥1 serves/day | 240 (98) | 277 (97) | 1.45 (0.52 – 4.04) |
| **Vegetable** |  |  |  |
| ≥2.5 serves/day | 91 (37) | 84 (30) | 1.39 (0.96 – 1.99) |
| **Fast food** |  |  |  |
| No | 91 (37) | 98 (34) | 1.15 (0.80 – 1.63) |
| **Soft drink** |  |  |  |
| No | 206 (83) | 243 (84) | 0.96 (0.61 – 1.53) |
| **Eating in front of TV** |  |  |  |
| No | 201 (81) | 199 (69) | 2.00 (1.33 to 2.99) P=0.001 |
| **Food for reward** |  |  |  |
| No | 201 (82) | 229 (79) | 1.17 (0.76 – 1.80) |
| ****Dietary behaviour*** |  |  |  |
| *Meeting all 6 recommendations above* | 33 (13) | 22 (8) | 1.89 (1.07 – 3.35) P=0.028 |
|  |  |  |  |
| **Outdoor play time** |  |  |  |
| ≥2 hours/day | 174 (70) | 183 (63) | 1.39 (0.97 – 2.00) |
| **Screen time** |  |  |  |
| <1 hour/day | 86 (35) | 92 (32) | 1.15 (0.80 – 1.66) |
| **Sleep duration** |  |  |  |
| ≥11 hours/day | 181 (73) | 192 (66) | 1.40 (0.96 – 2.03) |
| ****Physical activity/screen time/sleep behaviour*** |  |  |  |
| *Meeting all 3 recommendations above* | 58 (23) | 43 (15) | 1.76 (1.14 – 2.73) P=0.011 |
|  |  |  |  |
| **Mothers** |  |  |  |
| **Fruit consumption** |  |  |  |
| ≥2 serves/day | 127 (51) | 165 (57) | 0.80 (0.57 – 1.13) |
| **Vegetable consumption** |  |  |  |
| ≥5 serves/day | 33 (13) | 30 (10) | 1.34 (0.79 – 2.27) |
| **Physical activity time** |  |  |  |
| >150 minutes/week | 181 (73) | 224 (77) | 0.81 (0.54 – 1.20) |
| **Sedentary time** |  |  |  |
| ≤4 hours/day | 144 (58) | 163 (57) | 1.07 (0.75 – 1.50) |

Note: AOR: adjusted odds ratio, adjusted for original intervention allocation.

****Australian Institute of Health and Welfare 2020. Australia’s children. Cat. no. CWS 69. Canberra: AIHW***
